# Using a portable hydrogen cyanide gas meter to uncover a dynamic phytochemical landscape

**DOI:** 10.1002/aps3.11336

**Published:** 2020-04-19

**Authors:** John Smiley, Colin R. Morrison

**Affiliations:** ^1^ Office of Research Affairs University of California San Diego La Jolla California USA; ^2^ Department of Integrative Biology Graduate Program in Ecology, Evolution and Behavior The University of Texas at Austin Austin Texas USA

**Keywords:** cyanide gas detector, field measurements, hydrogen cyanide, La Selva Biological Station, *Passiflora* (Passifloraceae), phytochemical landscape

## Abstract

**Premise:**

Over 3000 species of plants and animals release toxic hydrogen cyanide (HCN) gas when their tissues are crushed. To investigate the role of cyanogenesis in *Passiflora*–herbivore interactions, we developed an inexpensive, rapid, sensitive method for measuring HCN emissions from crushed tissues.

**Methods:**

The method includes crushed tissue confinement in a closed chamber, where cyanogenesis reactions occur, followed by evacuation of gas to a portable HCN meter. Parts per million readings are repeated at 5‐min intervals until HCN is depleted. Three versions of the closed reaction chamber apparatus were tested: plastic cup, airtight combination mortar‐pestle, and glass desiccator jar.

**Results:**

We calibrated the method by comparing with a closed chamber measurement apparatus. The procedure's repeatability was demonstrated with a standard curve using known quantities of cyanogenic glycoside standard. Data collected with this method were also compared with the conventional colorimetric procedure. We processed over 2000 samples using this technique, revealing diverse elements of cyanogenic variation.

**Conclusions:**

These methods produced well‐defined data with minimal error. Results illustrated a one to four order‐of‐magnitude variation at organizational levels ranging from individual leaves to the entire *Passiflora* community. We now have a promising tool for uncovering the HCN phytochemical landscape in unprecedented detail.

A major component of terrestrial diversity is the landscape of toxic and repellent chemicals found in organisms (Hunter, 2016). In plants belonging to many families, crushing or chewing leaves releases potentially toxic hydrogen cyanide gas (HCN). Cyanogenesis has been recognized in over 3000 species of vascular plants distributed throughout 110 different families of ferns, gymnosperms, and both monocotyledonous and dicotyledonous angiosperms (Zagrobelny et al., 2008). These include a diverse array of economically important species used by humans such as cassava, stone fruits, lima beans, bamboo shoots, and cashews. Nearly all species of the plant genus *Passiflora* L. (Passifloraceae) are known to contain HCN‐releasing cyanogenic glycosides. Many *Passiflora* subgenera and subgeneric sections have unique forms of these compounds, as well as unique β‐glucosidase enzymes specific to each type of cyanogen (Spencer, 1988; Fig. 1). The compounds are also known to interact with herbivorous *Heliconius* butterfly larvae, possibly forcing specialization and contributing to species diversity (Gilbert, 1991; Pinheiro de Castro et al., 2018).

**Figure 1:**
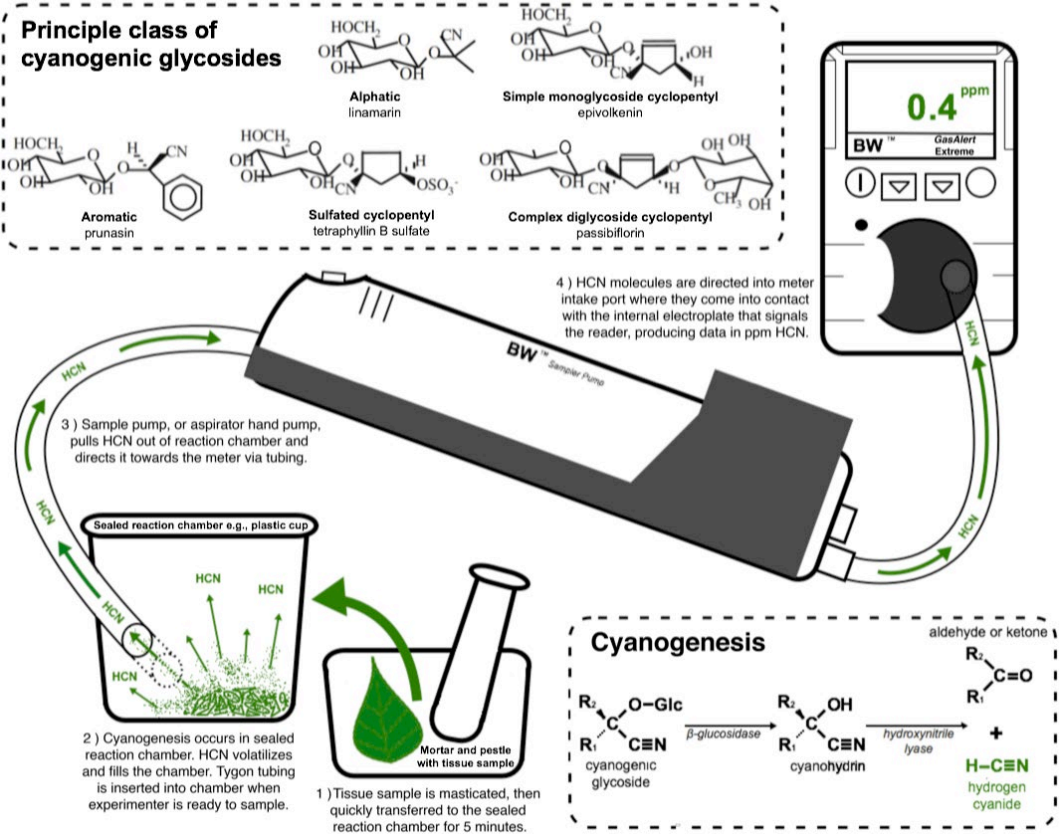
Annotated schematic of HCN gas measurement apparatus. The insert on top left shows the five principal classes of cyanogenic glycosides; the insert on bottom right shows the reactants and products involved in cyanogenesis of a typical cyanogenic glycoside molecule.

In 2012, we began surveying cyanogenesis in *Passiflora* at the La Selva Biological Station (La Selva) in northeastern Costa Rica (10**°**25′53″N, 84**°**00′17″W). This was part of a study of the natural history of *Passiflora*‐feeding herbivores, comparing flea beetle (Coleoptera: Chrysomelidae) and *Heliconius* butterfly (Lepidoptera: Nymphalidae) interactions with the plants and their unique, complex forms of cyanogenic glycosides and other chemicals (Smiley, 1985; Smiley and Wisdom, 1985; Spencer, 1988; Engler et al., 2000). To date, most analyses of cyanogenesis have been laboratory based, using greenhouse‐propagated plants and requiring expensive analytical equipment to quantify cyanogens (e.g., Schappert and Shore, 2000; Arias et al., 2016; Pinheiro de Castro et al., 2019). However, this study only required an inexpensive, rapid, field method for measuring HCN production from crushed *Passiflora* leaves. This was achieved by using a snap‐lidded plastic cup as a reaction chamber and measuring gaseous HCN with a portable meter developed for personnel working in potentially hazardous situations (Fig. 1).

The “plastic cup” technique was used on fresh plant material brought in from the field between 2012 and 2019. When larger leaf samples were involved, it was useful to place the HCN meter in a glass desiccator jar with the crushed leaf sample. HCN amounts could then be read by looking at the meter display through the glass wall of the jar (Appendix 1). Testing of these methods with more than 2000 measurements revealed a complex phytochemical landscape consisting of contrasting spatial and temporal patterns of cyanogenesis in different *Passiflora* species, individuals, tissues, and even among leaves distributed along branches. There was also evidence for dynamic adaptation by the plants in response to insect herbivory.

In 2017, we began using the plastic cup device to investigate cyanogenesis in *Passiflora* distributed throughout Texas and the southeastern region of the United States, resulting in the testing of more than 900 additional temperate samples. In 2019, we developed a nearly airtight combination mortar‐pestle (MP) reaction chamber to replace the plastic cup (Appendix S1). This unit has the advantage of combining the mortar and pestle crushing action and the reaction chamber into one closed apparatus. Moreover, it may limit the amount of HCN loss because the material can be thoroughly milled in a closed space with minimal breakage of the hermetic seal. The total cost of design, fabrication, and parts for the custom reaction chamber was low (ca. US$200/unit). The sensitivity was comparable to the plastic cup technique, and throughput was equally fast.

Below we describe the three methods in enough detail to replicate the apparatus and methodologies for the plastic cup, the glass desiccator jar, and the MP. We then evaluate the accuracy and precision of the techniques using five approaches: (1) by comparison with the conventional Lambert colorimetric laboratory technique (Lambert et al., 1975), (2) by comparing plastic cup to closed glass desiccator jar readings on carefully mixed leaf tissues, (3) by error analysis of selected data trends, (4) by validating repeatability of the procedure using commercial cyanogenic glycoside standard, and (5) by comparison with published quantities of cyanogenic glycosides in *Passiflora*. Finally, we present examples from our *Passiflora* field work that illustrate the power of these techniques to uncover previously unknown elements of the phytochemical landscape.

## METHODS

### Plastic cup device

The plastic cup device and procedure were developed to measure relatively low concentrations of HCN gas produced by crushing *Passiflora* foliage. Experiments began by recording time zero (t_0_) and crushing tissue for the first sample with mortar and pestle, and then processing two more samples in the same manner, resulting in three samples confined within sealed cups, each outgassing HCN into their respective reaction chambers. Measurements were repeated every 5 min until the concentration of gas decreased below the lower sensitivity limit of the meter (0.3 parts per million [ppm]); the resulting data then described the rate of HCN release as well as the total quantity released. See Appendix 2 for a detailed step‐by‐step description of the sampling protocol.

**Figure 2:**
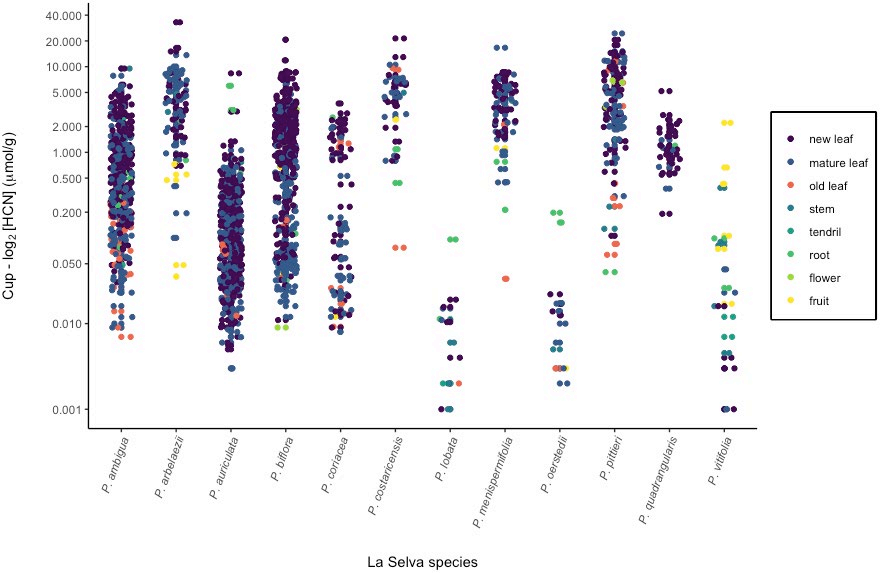
Total amount of HCN gas released, per gram wet weight of freshly crushed *Passiflora* tissues, for a total of 1808 measurements. The *y*‐axis is plotted on a log_2_ scale.

In general, the total HCN values are expected to be proportional to the amount of cyanogenic glycoside in the plant tissues as long as there is sufficient β‐glucosidase enzyme in the foliage to cause hydrolysis (H. Engler, personal communication). Changing the amount or activity of β‐glucosidase enzyme will alter the rate of release but should not affect the final quantity. A typical cyanogenesis reaction has been depicted in Fig. 1. The sample amount varied with the species being processed; 10 mg of tissue was sufficient for the more cyanogenic species (e.g., *P. pittieri* Mast.), whereas up to 300 mg was required, in some instances, for species with reduced HCN concentration (e.g., *P. auriculata* Kunth). Figure 2 depicts concentration of HCN in the tissues of La Selva *Passiflora* species.

Materials included a portable HCN meter (BW Technologies by Honeywell, Lincolnshire, Illinois, USA) designed for personnel working in potentially hazardous situations in which large quantities of cyanogenic material are present. These meters slowly lose sensitivity over time and use, and were therefore replaced after approximately two years of use. As a precaution, the same meter was used during series of samples to avoid minor differences in sensitivity. As a less expensive alternative to replacing the entire unit, replacement HCN electrostatic sensors are commercially available. Materials required for assembly of the HCN measurement apparatus include: The sampler gas pump with an accompanying connector device for attaching to the meter (BW Technologies by Honeywell), approximately 60 cm of 1/4‐inch outside diameter flexible clear polyvinylchloride (PVC) tubing, 1/4‐inch diameter drill bit, and three snap‐cap polyethylene airtight plastic cups with lids (Glad Products Company, Oakland, California, USA). A hand‐operated pump (Manual Aspirator Pump Kit plus Probe, BW Technologies by Honeywell) can be used in place of the battery‐powered sampler pump with equal accuracy and efficiency (C.R.M., unpublished data). The manual pump had a flow rate that was approximately equal to the sampler pump if the pump was compressed once per second. Several additional supplies are necessary to conduct the experiment: a milligram balance for weighing the cup and sample, a 5–10‐cm diameter mortar and pestle, tape to cover the hole in the plastic cup, and a clock. Total cost of parts for the apparatus was low (ca. US$650/complete unit). Refer to Appendix S2 for an image of the apparatus setup.

### Desiccator jar

The desiccator jar method is an alternative for measuring HCN gas production. Using this method, the HCN meter and the crushed sample are placed on the shelf of a 9.6‐L glass desiccator jar. The meter is placed so the display can be read through the wall of the jar (Appendix 1). Readings can be made at any desired interval; to monitor reaction kinetics in real time, we recorded at 20‐s intervals. Note that any closed container that is transparent and large enough to hold the HCN meter and the sample can be used. Because the chamber is closed, the ppm of HCN gas accumulates until it stabilizes. Molarity (μmol/g HCN) may be calculated as ([ppm · (chamber volume/22.4 L)]/sample weight). The advantage of this technique is that it is closed to the outside air with no dilution or loss, and thus provides an accurate measure of the ppm HCN generated. The disadvantage is that small samples combined with low concentrations of HCN fail to give positive readings because they are undetectable by the meter (<0.3 ppm).

### Combination mortar‐pestle chamber (MP)

We developed the MP device to test the efficacy of the plastic cup and dessicator jar methods, with the potential to select the method (i.e., plastic cup, dessicator jar, or MP) that resulted in the most improved data capture. The MP device has the advantage of combining the mortar and pestle crushing action and the reaction chamber into one closed unit. The reaction chamber is small (52 mL), which potentially increases the sensitivity when measuring small samples or those with low values of HCN. A description of the fabrication process for this apparatus is presented in Appendix S1.

Pre‐weighed plant material is placed in the depression at the bottom of the chamber and ground with a glass rod. The stopper is briefly removed to break the vacuum in the chamber, and the rod is retracted from the chamber to a set position so that volatile HCN can fill the chamber. When the user is ready to take a reading, the rubber stopper is removed, and the tube attached to the air pump is inserted into the side thread. The pump evacuates the sample air in the same manner as in the cup method. The removed air is replaced by removing the glass rod for 3 s. The rod is then inserted back into the chamber, and the rubber stopper is placed back into the thread once the reading has been recorded. Readings can be made at any desired interval as with the other devices (Appendix 2). Numerous internal threads or similar chamber‐like glassware could be utilized in similar closed MP chambers.

### Evaluation of the cup and MP procedures

Method validation was accomplished by dividing single leaf samples and running them with the new methods described above in parallel to the conventional Lambert colorimetric technique (Lambert et al., 1975). The hypothesis was that values of the new methods will be identical to or in a fixed ratio with the values revealed by the standard technique.

Material for the method comparison was harvested at La Selva in July 2019. Eight *Passiflora* species characterized by five classes of cyanogenic glycosides (Engler and Gilbert, 2007; Fig. 1) and belonging to three *Passiflora* subgenera (i.e., *Passiflora*,* Decaloba* (DC.) Rchb., *Astrophea* (DC.) Mast.) were sampled (3–5 individuals/species, *N* = 31 total samples). Leaf tips were quickly excised with clean scissors and cut into four approximately equal‐sized pieces. Two of these pieces were used for a comparison of the plastic cup method, and the other two were used for the MP comparison; leaf pieces for each comparison were from the same side of the leaf midvein. Two pieces were weighed and sampled immediately with the HCN meter. The corresponding pieces were weighed, placed in paper envelopes, and stored in the freezer at −80°C until HCN extraction. Values from pieces of an individual leaf that underwent the same measurement process (cup or colorimetry) were averaged for each plant sample.

### Cyanide extraction and quantification

The following extraction method is a modification of the Arias et al. (2016) protocol. Leaf material tissue was milled with liquid nitrogen and weighed. Total cyanogenic glycosides were extracted by adding 0.1 M H_3_PO_4_. The mixture was filtered and mixed with 5 M H_2_SO_4_ and heated at 98°C to hydrolyze the compounds. The solution was then cooled in an ice bath. Next, cold 5 M NaOH was added to trap the liberated cyanide molecules as NaCN.

Quantification followed the Lambert cyanide colorimetric method (Lambert et al., 1975) and was compared with the new HCN meter quantification method presented here. Aliquots of the final solution were added to 0.2 M phosphate buffer, and *N*‐chlorosuccinimide/succinimide oxidizing solution was added. Finally, chromogenic pyridine/barbituric acid solution was added. Absorbance of the samples was measured using a UV‐Vis spectrophotometer (Helios Spectronic Unicam; Thermo Fisher Scientific, Waltham, Massachusetts, USA) at 580 nm against a blank. NaCN concentration of each sample was calculated by comparing its absorbance with a calibration curve of solutions with known NaCN concentrations (*R* ^2^ = 0.99, *P* < 0.001). See Appendix 3 for the detailed HCN extraction and colorimetric quantification protocol.

HCN concentration values (μg/g dry weight) were converted to μmol/g so that units were comparable with the concentration data from the meter method. Data from the pairwise cup–MP comparisons (one comparison per sample) were analyzed with linear regression. The fixed ratio hypothesis was tested by plotting log_10_ of the new method values against log_10_ of the colorimetric method and calculating the slope (slope = 1 if the ratio is constant) and *y*‐intercept (10^b^ = ratio). All statistical analyses were conducted in the R computing environment version 3.5.1 (R Core Team, 2018).

### Calibration of the plastic cup technique

The desiccator jar method uses a closed volume to accurately measure total emission of HCN gas from crushed samples. We used this property to calibrate the plastic cup values as follows: Using a new, calibrated HCN meter, we quickly homogenized *P. biflora* Lam. leaves and evenly partitioned the tissue into two subsamples. One sample was measured following the cup protocol mentioned above (details in Appendix 2); the other was placed inside a sealed glass desiccator jar (Appendix 1) where HCN was measured until meter readings reached an asymptote. We compared the desiccator jar values to the plastic cup values by plotting log_10_‐transformed HCN values on a graph and fitting a straight line to the data. A fixed ratio was calculated as described above. See Appendix 4 for the detailed procedure on how we processed the tissue for this experiment.

### Error evaluation in series of related data

We selected three sets of related data that illustrate the precision and repeatability of the plastic cup technique. Each data set consisted of leaves sampled from a *Passiflora* branch (*N* = 2 species) ranging from the newest leaf at the branch tip (leaf #1) to older leaves (up to leaf #20). Leaves for each branch were measured within a 2‐h period. HCN concentration was plotted as a function of leaf position along the branch, and then fitted with a fourth‐order polynomial curve to the data. The root mean square error was calculated and compared to the data range to yield average error value as a percentage of the total.

### Calibration of cup and MP data with a standard curve

We purchased a commercially available cyanogenic glycoside, amygdalin (Sigma Aldrich, St. Louis, Missouri, USA), and created a standard curve using known quantities of amygdalin and corresponding HCN data in parts per million. The goal of this experiment was to verify that the HCN meter apparatus functioned as expected with known standard quantities, and to present a standard curve that experimenters can use in their studies. Cyanogenesis was accomplished endogenously by adding 100 μL of stock β‐glucosidase solution (10 mg/mL) to a series of 1‐mL amygdalin dilutions. This enzyme hydrolyzes the HCN molecule from the glucose chain, liberating it as a measurable gas (Fig. 1). The cyanogen‐glucosidase solution was mixed with a pipette for 5 s, and then immediately placed inside the plastic cup or the MP reaction chamber. HCN data collection followed the procedure described above.

### Comparison with published data

Mean species HCN quantities from the plastic cup method were compared with a previously published data set on *Passiflora* cyanogenic glycoside concentration (Engler and Gilbert, 2007). These investigators used the Lambert colorimetric method to quantify HCN content of their samples (Lambert et al., 1975). Linear regression was used to account for the percent variation explained by species HCN concentration between the two studies. A paired *t*‐test was also conducted to assess whether there was a statistically significant difference in mean species HCN values between the two studies.

### Calculating duration of HCN release

In order to compare reaction kinetics, the duration of HCN release was calculated for each sample as a weighted mean. For the *n*‐th meter reading in a *Passiflora* sample with *N* positive readings, the time since tissue crushing (t_n_–t_0_) was multiplied by the corresponding amount of HCN shown on the meter (ppm_i_). These *N* products were summed over all positive values and divided by the total ppm summed over *N* samples. This yielded the weighted mean time from crushing to release. This measurement produced a minimum value of 5 min because that was the first sampling time in our procedure. These mean times were then averaged for each species with cyanogenic foliage (Table 1).

**Table 1:** Duration of HCN release (in minutes) from crushed tissues in La Selva *Passiflora* species.^a^

Species	Mean	SD	Range	*N*
*P. biflora*	5.8	1.2	10.6	275
*P. ambigua*	6.2	2.8	42.5	314
*P. pittieri*	6.4	1.9	16.1	88
*P. auriculata*	6.7	1.8	12.7	329
*P. costaricensis*	6.9	1.5	5	23
*P. arbelaezii*	7.4	3.5	23	43
*P. menispermifolia*	7.4	4.6	22.1	31
*P. quadrangularis*	9.1	3.5	12	39
*P. coriacea*	39.6	21.5	80	39

a Minimum value of 5 min (first sample takes place at 5 min after crushing).

## RESULTS

### Validation study

#### Comparison with laboratory colorimetric technique

In the method comparison study, comparing sides of the leaf sampled by the spectrophotometer method had a positive relationship, accounting for approximately half of the variance (df = 33, *R* ^2^ = 0.54, *P* < 0.001; Appendix S3). This comparison was made as an exercise in proof of concept, with the prediction that colorimetric data taken from opposite sides of the same leaf should have a strong, positive, linear relationship. Overall, 54% variance explained by the relationship is relatively low considering that the data points came from the same leaf. In fact, colorimetric data from the same leaf explained less variation than colorimetric comparisons made with the plastic cup or the MP apparatus on the same side of a leaf, respectively (63% and 72%). There are at least two possibilities that could explain why the intra‐leaf colorimetric comparison was weak. One is that the HCN extraction and Lambert colorimetric procedures have low repeatability. Both protocols involve a variety of different solutions, take a long time, and require many preparatory steps in which systematic error could be introduced. The other possibility is that *Passiflora* intra‐leaf HCN variation is considerable. We have observed examples of such heterogeneous HCN content within individual leaves, using the plastic cup method on a single leaf of *P. ambigua* Hemsl. (Appendix 5). It is possible that both of these factors affected this result.

**Figure 3:**
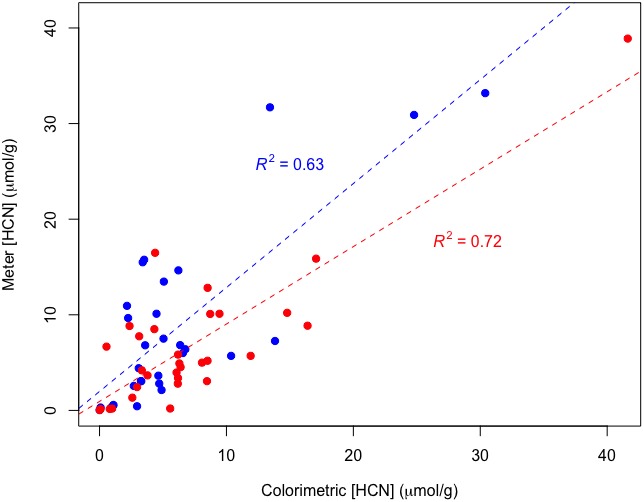
Comparison of HCN measurement methodologies. Plastic cup measurements as compared with laboratory colorimetric measurements are depicted in red; mortar‐pestle chamber measurements compared with colorimetric measurements are in blue.

The HCN concentration as measured using the plastic cup method had a statistically significant positive relationship with colorimetric HCN data sampled from the same subsection of an individual leaf (df = 32, *R* ^2^ = 0.63, *P* < 0.001; Fig. 3). This experiment explained 9% more variance than the colorimetric comparison of samples from opposite sides of the same leaf (Appendix S3). Mortar‐pestle HCN concentration data also had a significant, positive relationship with the colorimetric HCN data sampled from the same subsection of an individual leaf (df = 32, *R* ^2^ = 0.72, *P* < 0.001; Fig. 3), showing an 18% increase from the colorimetric comparison of samples from opposite sides of the leaf (Appendix S3).

The ratio between the plastic cup and colorimetric methods was modeled by regressing log_10_‐transformed cup values against the corresponding log_10_‐transformed colorimetric values. The observed *y*‐intercept value of 0.16 corresponds to a cup : colorimetric ratio of 1.45 (because 10^0.16^ = 1.45). The slope value of 0.87 ± 0.12 SE was not significantly different from 1 (*P* < 0.001), demonstrating a fixed ratio between the methods.

#### Desiccator jar and plastic cup HCN measurement calibration

**Figure 4:**
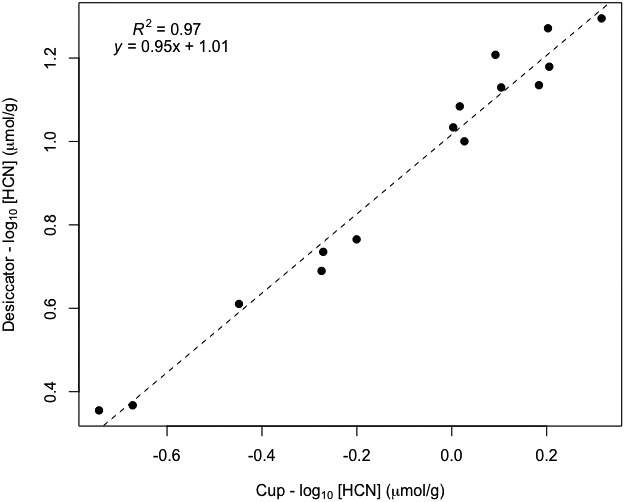
Calibration of plastic cup values by comparison with *Passiflora biflora* leaf measurements inside a completely closed desiccator jar. Values have been log_10_‐transformed to even out the variance. The fitted line slope of 0.95 is not significantly different from 1, indicating a nearly constant ratio of cup‐to‐jar values, and the *y*‐intercept of 1.01 indicates a ratio of 10.2. This means that the cup values should be multiplied by 10.2 to achieve accurate HCN concentrations.

We calculated the ratio between the plastic cup and desiccator jar methods as described in the previous section. The linear equation revealed a cup : desiccator ratio of 10.2 (Fig. 4). Again, the slope was not significantly different from a value of 1 (0.95 ± 0.04 SE, *P* < 0.001), which is consistent with the hypothesis of a fixed HCN ratio between the measurement methods.

#### Estimated measurement error using the plastic cup method

Measurement error of the method was generated by using the plastic cup technique to look at leaf cyanogenesis along three branches of *Passiflora* (Table 2). Error was estimated by fitting a fourth‐order polynomial curve to the data, calculating the root mean square error of points around the curve, and dividing the error by the data range. In these samples, entire *P. biflora* or *P. auriculata* leaves were taken for analysis. In each case, the error was between 2–3% of the data range, indicating that the plastic cup method is repeatable, capturing HCN variation with a high degree of precision.

**Table 2:** Plastic cup measurement precision, as estimated from cyanogenesis measurements of leaves spaced along three branches of *Passiflora*.^a^

Data curve source^b^	Species	Data curve label	*N*	*R* ^2^	RMSE	Curve range	Error % curve range	*P* value
Appendix S4	*P. auriculata*	48 h alt leaf	6	0.99	0.019	0.7	2.8	0.0002
Appendix S5A	*P. biflora*	All data points	11	0.99	0.306	12	2.5	0.0001
Appendix S5B	*P. biflora*	14 h cut leaf	8	0.99	0.137	5	2.7	0.0001

Note

RMSE = root mean square error.

a A polynomial curve was fitted to the data, and the RMSE calculated. This error, divided by the data range, yielded a value between 2.5% and 2.8% in all three cases.

b Data are visualized in the Supporting Information as noted.

#### Calibration of plastic cup and MP data with a standard curve

**Figure 5:**
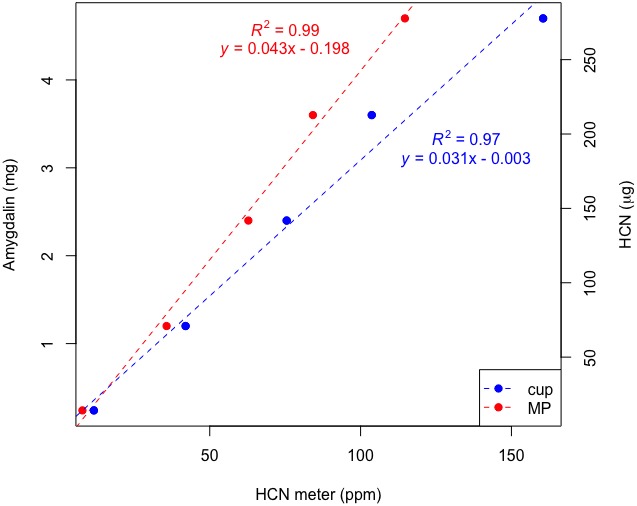
Standard curve of amygdalin (mg)/HCN (μg) on the *y*‐axes vs. ppm HCN on the *x*‐axis. Data for the cup and the mortar‐pestle (MP) are plotted here. Dotted lines are best‐fit lines.

The standardized range of amygdalin mass had a very strong relationship with ppm HCN meter data for the plastic cup (df = 4, *R* ^2^ = 0.97, *P* < 0.0001) and the MP (df = 4, *R* ^2^ = 0.99, *P* < 0.0001) methods, resulting in two standard curves with tight fit (Fig. 5). Slightly different linear relationships were the result of the different reaction chamber volumes between the plastic cup (0.143 L) and the MP (0.052 L). See Fig. 5 for the plastic cup and MP linear equations. The amygdalin range of 0.2–4.7 mg (14.18–277.73 μg HCN) corresponded with 8–171 ppm HCN.

### Comparison with published data

**Figure 6:**
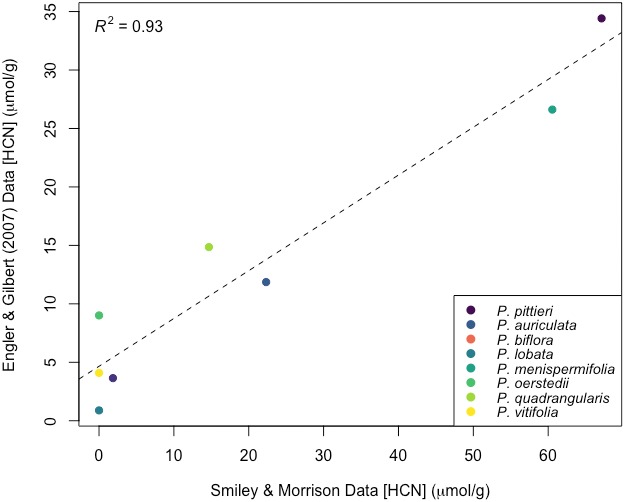
Relationship between 142 measurements of foliar cyanogenic glycoside HCN, published in Engler and Gilbert (2007), and 531 plastic cup HCN measurements at La Selva Biological Station. Means were calculated for each *Passiflora* species after converting units to μmol/g fresh weight and applying a calibration of 10× to the plastic cup values. Data used with permission from H. Engler and L. Gilbert.

Engler and Gilbert (2007) provided measurements of cyanogenic glycosides in many of the same *Passiflora* species as found at La Selva. Converting from their published μg/g dry weight units to μmol/g fresh weight by a factor of 10, we compared mean HCN quantities for each species (Table 3). A close fit was found between the plastic cup and colorimetric data (df = 7, *R* ^2^ = 0.93, *P* < 0.001; Fig. 6). A paired *t*‐test confirmed our hypothesis that there was no statistically significant difference between mean species HCN values across the data sets (df = 7, *t*‐test = −1.29, *P* = 0.24). Colorimetric laboratory mean species values ranged from 46% to 100% of the calibrated plastic cup values reported here.

**Table 3:** Comparison of plastic cup field measurements to values published in Engler and Gilbert (2007). Laboratory values were converted from μg/g dry weight units to μmol/g fresh weight, and field values multiplied by the calibration factor of 10 (see Methods). This root mean square error is approximately 10% of the measured data range (RMSE = 3.4). Three species, *P. lobata*,* P. oerstedii*, and *P. vitifolia*, were acyanogenic by the plastic cup method, but contained cyanogenic glycosides as measured in Engler and Gilbert (2007).

*Passiflora* species	Published HCN μg/g dry foliage		Plastic cup measurements at La Selva		Converted published data mean	Calibrated plastic cup mean
	Mean	*N*	Mean	*N*	HCN μmol/g fresh weight	
*P. pittieri*	4474	10	6.582	71	34.42	67.13
*P. auriculata*	474	32	0.183	302	3.65	1.86
*P. biflora*	1542	25	2.188	303	11.86	22.32
*P. lobata*	115	8	0.000	4	0.88	0.00
*P. menispermifolia*	3460	7	5.936	25	26.62	60.55
*P. oerstedii*	1171	18	0.000	8	9.01	0.00
*P. quadrangularis*	1931	22	1.439	38	14.85	14.68
*P. vitifolia*	532	20	0.000	8	4.09	0.00

### Field investigations

The desiccator jar, plastic cup, and MP methods were used in the field between 2012 and 2019, generating over 2200 measurements. The low cost and rapid throughput of these methods enabled us to carry out new types of field investigations. We present some highlights of these results below.

We investigated intra‐ and interspecific heterogeneity in cyanogenesis for *Passiflora* vines at many different scales ranging from species to individual plant tissues. Four‐order‐of‐magnitude variation was found in foliage cyanogensis (Fig. 2), with four species consistently highly cyanogenic (2–15 μmol/g), two species moderately cyanogenic (0.5–5 μmol/g), two species with highly variable amounts (0.1–10 μmol/g), one species with low but highly variable HCN (0.01–1 μmol/g), and three species with no measurable amounts. We also measured different plant parts in addition to foliage, such as fruits, stems, flowers, and roots (Fig. 2). In some cases, these were quite different from foliage measurements in the same species (Fig. 2). Two species in particular showed opposing trends: *P. biflora* had highly cyanogenic foliage with relatively acyanogenic roots, whereas *P. auriculata* had more HCN in the roots than in the foliage. The three other species mentioned above with acyanogenic leaves did have cyanogenic roots, stems, and fruit. Additionally, we measured variation within single leaves of *P. ambigua* (Appendix 5) and found three‐fold variation in different parts of the same leaf.

The three most variable *Passiflora* species indicated in Fig. 2 (*P. auriculata*,* P. biflora*, and *P. ambigua*) all displayed great variability among and within individual plants. This intraspecific variation is especially well defined in *P. auriculata* (Appendix S4). Ten‐fold differences were found among individual plants (e.g., SP4A and SP4D), and branches within plants were also found to vary greatly (see SP1C). We also measured HCN from leaves along branches starting at the newest full‐sized leaf, observing a clinal decrease with leaf age in *P. coriacea* Juss., *P. ambigua*, and *P. quadrangularis* L. Conversely, we found an increase with leaf age in other species: *P. auriculata*,* P. costaricensis* Killip, *P. menispermifolia* Kunth, and *P. pittieri*. A decrease and an increase in HCN with leaf age were observed in separate *P. biflora* experiments (Appendix S5).

Mechanical leaf damage did affect *P. ambigua* cyanogenesis within days after leaves were cut (Appendix S6A). Cutting tips off the odd‐numbered leaves caused an apparent doubling of HCN 24–48 h later, but in those leaves only. Adjacent leaves showed no response to the treatment of their neighbors; in fact, these leaves showed no response to the 24‐h sampling of adjacent leaves at 48 h, suggesting that cyanogenesis was suppressed in leaves that had not experienced damage. By contrast, *P. auriculata* showed no evidence that cyanogenesis would increase after leaf tip sampling. The plastic cup technique has the power to resolve small differences in cyanogenesis among the tissues of individual plants. This power is exemplified with *P. biflora* leaves displaying stability in the presence of sampling over a 54‐h period (Appendix S5A). Plucking leaves at the petiole appeared to have no effect on adjacent leaves, within an error of only 2.5% (Table 2).

**Figure 7:**
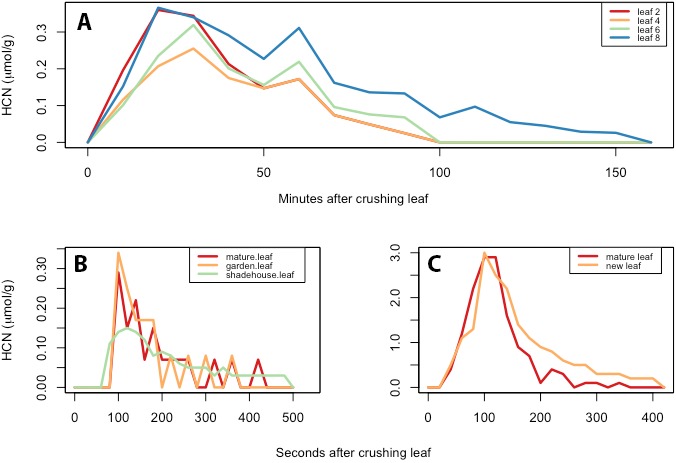
HCN reaction kinetics in three *Passiflora* species. (A) Extremely slow HCN reaction kinetics in *Passiflora coriacea*, requiring 1–2 h to reach 95% depletion. (B, C) Kinetics of HCN gas release by crushed foliage of two species, *P. ambigua* (B) and *P. menispermifolia* (C), in a closed desiccator jar apparatus. Peak release occurs at 120–130 s after crushing, and 90% depletion is reached within about 300–400 s. This is the most common pattern of HCN release, observed in most species of *Passiflora* at La Selva.

*Passiflora* species collected at La Selva display two principal HCN kinetic patterns— “slow” and “fast” release (Fig. 7). Comparing rates in Table 1 shows minimal variation in HCN release kinetics among the species tested, except for the very long‐duration reactions in *P. coriacea* (Fig. 7A). The only other species with a suggestion of slow HCN release is *P. quadrangularis*; this species may possess a dual‐release mechanism in which most release happens in the first few minutes, but then is followed by slow release. The higher resolution (20‐s interval) fast reactions in *P. ambigua* and *P. menispermifolia* (Fig. 7B, C) were duplicated with similar fast reactions peaking at about 120 s in all species tested except *P. coriacea*.

The meter methods were capable of capturing data that described dynamic patterns of HCN variation in an empirical context as well. *Passiflora* tissues have the ability to alter patterns of cyanogenesis rapidly and dynamically in response to tissue damage (Appendix S7). Cutting a branch at leaf #14 and returning it to the lab resulted in a 50% reduction in cyanogenesis along the branch in just 12 h (compare red and blue points). By 24 h, in the remaining live branch, only traces of HCN were detected (black points). This method was also used to quantify cyanogenesis in response to herbivory by group feeding *Heliconius doris* (L.) caterpillars (Lepidoptera: Nymphalidae) as measured by the plastic cup technique in leaves of *P. ambigua* (Appendix S7). Discs were sampled from leaves hosting large groups of feeding *H. doris* larvae (50*–*100 individuals) and from adjacent intact leaves that had not experienced herbivory. Larval feeding appeared to suppress cyanogenesis by factor of 3.

## DISCUSSION

### Validation study

The amygdalin standard calibration presented here demonstrates that our method is repeatable and capable of capturing a wide range of HCN concentrations. Almost 100% of the variance was explained by the linear relationship between standard mass and HCN meter measurements in parts per million for the plastic cup and the MP. The slight departures from a perfect linear relationship are likely due to systematic error such as minor variations in standard mass, slight differences in time the reaction chamber was evacuated after measurements, or chamber air tightness. Users could plot ppm HCN (measured) vs. ppm HCN (expected from standard) to ascertain whether such sources of error are affecting repeatability. The standard curves provide reference equations that experimenters can use to calibrate meter data and determine the mass of HCN molecules in a sample of known cyanogenic glycoside source. In other words, milligrams of HCN in a sample can be calculated by determining the ratio of HCN to amygdalin (or other known cyanogen) and substituting a ppm HCN value for *x* into the equations provided on Fig. 5. For example, amygdalin has a molecular mass of 457.4 g/mol, which corresponds to 27.03 g/mol HCN; therefore, 1.0 mg of amygdalin contains 59.09 μg HCN and corresponds with 32.34 ppm HCN. Users should calculate a new calibration curve if employing a cyanogenesis reaction chamber with a different volume than what was utilized here. In this case, the volume of the reaction chamber would be measured and inserted into a dimensional analysis equation that begins with measured ppm values in units of mg/L (1 ppm = 1 mg/L). A series of dimensional analysis equations solved for different ppm HCN concentrations generates data to calculate a new calibration curve into which users can insert ppm values to calculate their corresponding HCN mass values. Experimenters can purchase commercially available cyanogenic glycoside standards to perform this validation, including, but not limited to, prunasin, amygdalin, dhurrin, and lotaustralin.

One problem reporting results from the laboratory colorimetric validation measurements was the lack of a “uniform leaf” containing consistently homogenous amounts of cyanogenic glycosides. This issue is likely exacerbated by laboratory sampling procedures with the potential to introduce systematic error to the data. The fact that two colorimetric measurements from the same leaf only explained 54% of the variation between samples indicates that intra‐leaf variation and low repeatability of the sampling method are potential confounding issues that may act synergistically to produce a weak relationship between samples that were expected to covary with each other. These unknowns make it difficult to accurately compare the new methods to the conventional technique (Appendix S3).

The plastic cup and MP comparison with colorimetric data from the same subsection of a leaf explained 9% and 18% more variation, respectively, then the comparison of colorimetric data from opposite sides of the same leaf. Some of this difference can be attributed to *Passiflora* intra‐leaf variation (Appendix 5), and we infer that questionable repeatability of the extraction and colorimetric methods also affected this result. Regardless of error stemming from the method, these data suggest that HCN variation is greater between sides of a leaf than among samples from the same side. Together, these results indicate that the new methods are as effective at capturing natural HCN variation, if not more so, than the Lambert protocol.

Thirty to fifty percent of samples measured with the meter exhibited higher HCN concentration than the corresponding colorimetric sample in the method comparison experiment. Because the colorimetric concentration value theoretically captures all the available NaCN in a sample, this result may reflect variation within the original leaf sample as already discussed, or it could signify improvement in HCN detection ability by the meter methods over colorimetry in some samples. The leaf processing and extraction processes that proceed the colorimetric HCN quantification involve many steps where minor variations from a precise laboratory workflow could result in some loss of HCN. This potentially problematic situation is alleviated with the straightforward sampling procedure presented here, in which many samples can be processed and measured quickly.

The desiccator jar vs. plastic cup comparison provided a clearer way to accurately calibrate the cup method. The plastic cup method consistently measured 10% of the value obtained using the closed desiccator jar, yielding a calibration constant of 10.2 (Fig. 4). Engler and Gilbert (2007) provide measurements of cyanogenic glycosides in many of the same species as found at La Selva, and these are compared in Table 3. This relationship was visualized in Fig. 6 after multiplying the plastic cup values by 10, the calibration constant calculated above. The plastic cup measurements from La Selva are approximately double that of the colorimetric laboratory measurements from Engler and Gilbert (2007) in the aggregate, but this approximation varies among species. Multiplying the mean plastic cup data for each species by the calibration constant calculated here aligned the values from these studies to near equality. Factors such as differences between field and greenhouse‐grown plants or differences in sampling (e.g., leaf age) may have contributed to discrepancies between some of the species’ means.

### Field investigation of the *Passiflora* phytochemical landscape

The new measurement techniques presented here will be of great utility in uncovering diverse elements of the phytochemical landscape. Using this method, we found major variation in cyanogenesis among the 12 *Passiflora* species at La Selva, with variation ranging over four orders of magnitude among species and one to two orders of magnitude within a single species (Fig. 2). Species differed greatly and could be divided into five groups based on the amount and variability of HCN in the foliage (see Results for details).

There was significant among‐plant, among‐branch, and within‐branch variation (Appendix S6). This suggests the possibility that *Passiflora* dynamically reallocate their cyanogenic resources, as they do their extrafloral nectar resources (Izaguirre et al., 2013), in response to various stimuli such as light and herbivore damage. We present possible evidence of such rapid dynamism (see Appendix S5B), as a possible explanation might be that trauma caused by branch cutting resulted in elimination of cyanogenesis in the surviving leaves. We also found increases in HCN release after artificial damage in some cases but not in others (Appendix S5); in fact, leaf damage may induce reduction in cyanogenesis (Appendix S7). Although at first this may seem counterintuitive, this type of reduction may be beneficial in plants being attacked by specialist herbivores (see review by Ali and Agrawal, 2012). It may result in lower levels of standing cyanogenic compounds in the leaves for larval herbivores to sequester. This could make the herbivores more vulnerable to natural enemies, slow their growth rates, or make the plant less attractive for oviposition.

The reaction rate analysis in Table 1 illustrates yet another unexpected aspect of the phytochemical landscape: existence of slow HCN release (avg. 120 min in *P. coriacea*) vs. the fast (avg. 5–10 min) HCN release that is typically found in the other species tested. The mechanisms are unknown but could possibly involve a mismatch or interference among ß‐glucosidase enzymes (Spencer, 1988). *Passiflora coriacea* has been found to contain several different types of cyanogenic glycosides (e.g., epivolkenin, passicoriacin, and other tetraphyllin B sulfate epimers; Spencer and Seigler, 1987; Pinheiro de Castro et al., 2019). This difference could have profound effects upon potential herbivores. Seemingly acyanogenic slow‐reacting foliage might escape detection by searching butterflies or beetles yet remain toxic to herbivores ingesting the leaves.

From 2018 to present, we have used the plastic cup apparatus and methodology to survey HCN from hundreds of individual plants belonging to 12 species (Appendix S8) of *Passiflora* at La Selva and six species native to Texas and the southeastern United States (i.e., *P. affinis* Engelm., *P. foetida* L., *P. filipes* Benth., *P. incarnata* L., *P. lutea* L., *P. tenuiloba* Engelm. ex A. Gray). These data are now being used to characterize the *Passiflora* phytochemical landscape, which will be compared with soil nutrient profiles and trophic interactions (C.R.M., unpublished data). Moreover, this technique is being used to measure HCN in cyanogenic arthropods such as *Heliconius* larvae, flea beetle larvae and adults (Appendix S9), as well as in *Passiflora* roots, stem, tendrils, flowers, and fruits (Appendix S8). The sensitivity of the technique is useful for measuring cyanogenesis in such small quantities.

Our results using the reported method illustrate the dynamism and spatial heterogeneity that seems to characterize this phytochemical landscape. The underlying mechanism and ecological significance of these phenomena are waiting to be investigated and discovered. The ability to make these findings was absolutely dependent on possession of a low‐cost, fast‐turnaround, fast‐throughput measurement system such as that employed here. More importantly, this technique can be applied in basic and applied contexts to much more than just passifloraceous taxa. There are several thousand vascular plant species representing at least 110 families that contain cyanogenic compounds, as well as a huge variety of arthropod taxa that exploit them. This technique has great potential to increase the efficiency and reduce the cost of characterizing this diverse assemblage of organisms.

## AUTHOR CONTRIBUTIONS

J.S. conceived the idea of using the gas detector as a measurement device and developed the sampling techniques. J.S. and C.R.M. measured cyanogenesis in *Passiflora* at La Selva Biological Station. J.S. and C.R.M. analyzed data and wrote the manuscript. C.R.M. made the figures and conducted laboratory analytical and validation studies.

## Supporting information


**APPENDIX S1.** Combined mortar‐pestle (MP) device.

**APPENDIX S2.** HCN gas measurement “plastic cup” apparatus showing benchtop setup at La Selva Biological Station, Costa Rica.

**APPENDIX S3.** Colorimetric data comparison.

**APPENDIX S4.** An example of several dimensions of *Passiflora* intraspecific HCN variation.

**APPENDIX S5.** Dynamic spatial and temporal HCN variation in *Passiflora biflora*.

**APPENDIX S6.** Possible within‐leaf induction of cyanogenic glycosides in *Passiflora ambigua* and *P. auriculata*.

**APPENDIX S7.** *Heliconius doris* (Lepidoptera: Nymphalidae) feeding‐induced reduction in cyanogenesis in *Passiflora ambigua*.

**APPENDIX S8.** Tabulation of sample sizes from Fig. 2.

**APPENDIX S9.** The plastic cup technique can also be used to measure cyanogenesis in crushed arthropods. Insects of each species were pooled into one sample to increase the likelihood of HCN detection.

## Figures and Tables

**Figure 8:**
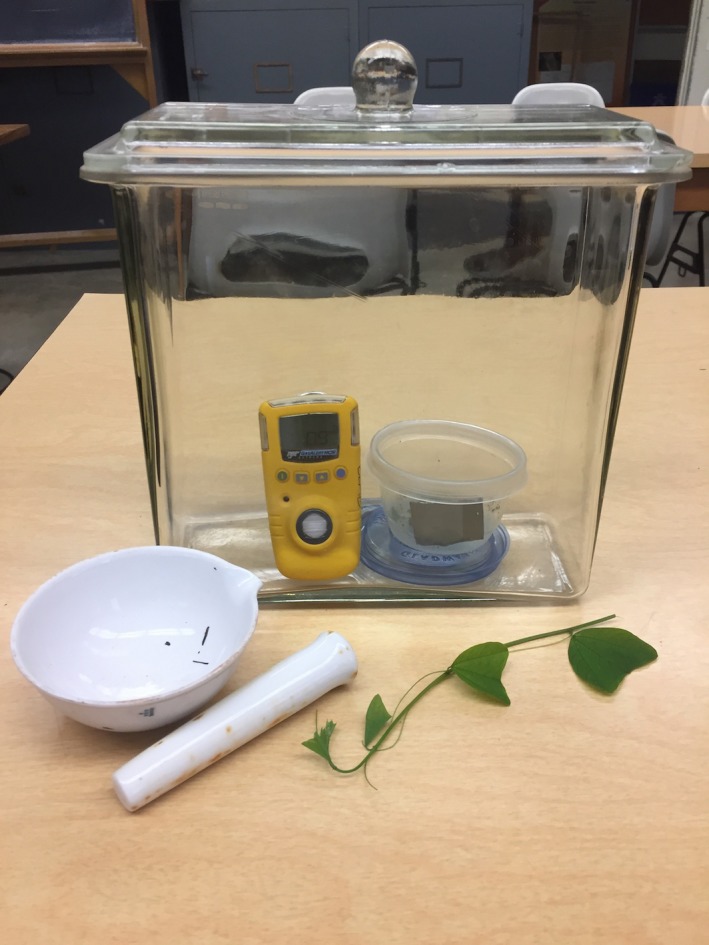
Detailed leaf processing and data collection procedure for the plastic cup technique.

**Figure 9:**
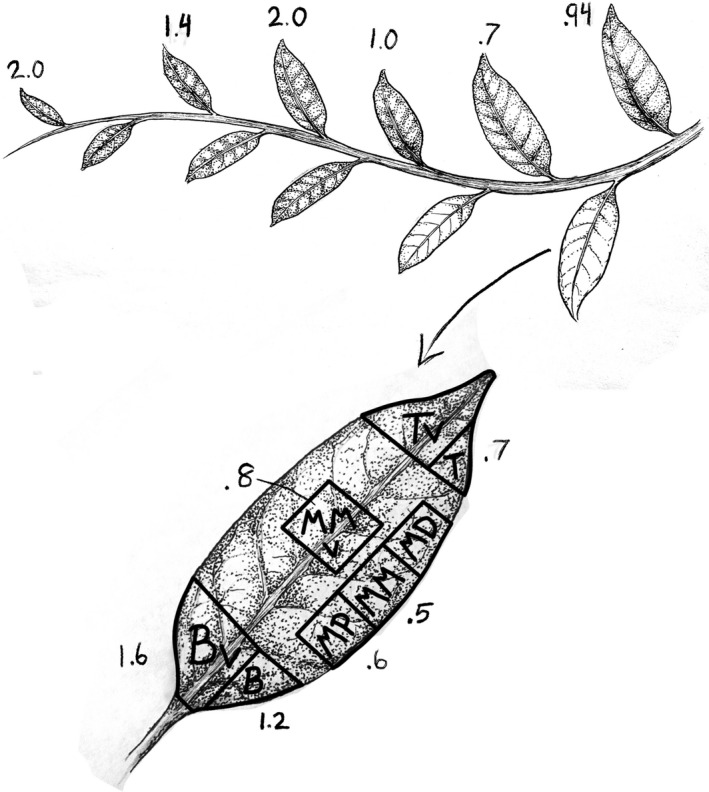
Illustration of HCN heterogeneity within an individual *Passiflora ambigua* branch and leaf. This illustration showcases one example of using the cup method to measure cyanogenesis along a branch and within a single leaf (μmol HCN/g fresh leaf). There is substantial variation, including possible trends for decreasing amounts as leaves age, more cyanogenesis toward the base of the leaf as compared with the middle and tip, and more HCN associated with the midvein of the leaf.

## Data Availability

Scripts and data for all analyses and plots in this contribution are available at https://github.com/cmorrison1/HCN-meter-procedure.

## APPENDIX 1

Image of closed chamber desiccator jar experiment with meter. HCN gas measurement using the closed chamber desiccator jar apparatus, showing the crushed sample enclosed with HCN meter. No gas can escape, and the meter measures the full amount of HCN produced.

## APPENDIX 2

### Preparation of apparatus

The procedure described below is for measuring HCN in three different leaves along a branch; it can be modified for other materials or as needed. Running three samples simultaneously was found to be optimal for maximizing throughput and maintaining accuracy and reliability. Using a hot, 1/4‐inch (6‐mm) drill bit, a hole is melted in the side of the plastic cups (3) and then covered with a small piece of tape. label the cups and corresponding lids 1–3, and measure and record the tare weight of cup, lid, and tape combination.

### Procedure

Collect three leaves from the plant and bring to the measurement area; use plastic bags to prevent drying if needed. Connect the sampler pump to the HCN meter and turn on the meter. Use clean scissors to cut off an appropriate amount of leaf material and place by sample cups 1–3; record sample info, cup number, and tare weight for each cup.

Record time zero (t_0_), and immediately begin processing the first sample, as follows: Gently fold the leaf into as small a bundle as possible (<1 cm^2^), hold the bundle in the bottom of the mortar with your fingertip, and crush by pushing down with the pestle. Once the bundle is pinned under the pestle, rock it back and forth just hard enough to crush the leaf parenchyma. The goal is to crush the leaf parenchyma, but not to tear up the fibrous structure of the leaf. After crushing, quickly remove the sample pieces and put them in cup #1. Snap on the lid and wait. At time = t_0_ + 1 min, bundle, crush, and place sample #2 in cup #2 and wait. At time = t_0_ + 2 min, bundle, crush, and place sample #3 in cup #3. There are now three samples in sealed cups, each outgassing HCN into the plastic chamber confined within the cup. Weigh the three cups, and record the gross weight of each on the data form. Then turn on the pump and leave it running.

At time = t_0_ + 5 min, peel off the tape from cup #1 and insert the tube from the sampler pump. It should fit snugly with no visible air leaks. Watch the meter; the readings start low and build over 10–20 s, reaching a peak value. Record the peak value on the data form next to the time. Then quickly remove the tube, replace the tape over the the cup hole, open the cup lid, and air out the cup for 3 s. Replace the lid and wait. At time = t_0_ + 6 min, repeat with the next cup (sample #2 in cup #2), and repeat with cup #3 at t_0_ + 7. The 1‐min interval is usually enough time for the meter to return to zero; if not, add extra seconds to the interval until zero or small values (e.g., 0.3–0.5 ppm) are reached. The following readings can then be shortened accordingly, going back to the original schedule of 5‐min intervals.

Repeat the three‐sample measurement starting at 10 min past time zero (t_0_ + 10), 15 min past (t_0_ + 15), and so on at 5‐min intervals until the ppm readings drop below 0.3 ppm. Terminate measurements when the peak ppm drops below 0.3. To calculate μmol HCN in a sample, multiply the sum total ppm for that sample (= ppm_1_ + ppm_2_ + ppm_3_ +… + ppm_n_), times the volume of the sample chamber, divided by the volume of one mole of gas (22.4 L under ideal conditions 25°C and 760 mm Hg atmospheric pressure). This value is then divided by the sample weight in grams to give μmol/g fresh tissue (total ppm × 0.00638 / sample weight). Our measurements were conducted at 25°C, at an altitude of 0 m above sea level, in laboratories at La Selva and the University of Texas at Austin. When operating under different temperatures, or at a different altitude, the experimenter should use the ideal gas law equation (PV = nrt) to adjust the mole volume constant in the formula. The mass of fresh plant tissue can be converted to the approximate dry weight by weighing, drying, and reweighing dried plant material from the same sample (e.g., tissue subsample from same leaf) or from the same *Passiflora* species.

### Technical note

The 60‐cm‐long, 1/8‐inch inside diameter tubing has a volume of about 5 mL, and the sampler pump and the manual aspirator move 5 mL/s; therefore, the transit time through the pump to the meter is about 1 s. As the cup evacuates, replacement air is forced in from gaps around the tube opening, mixing with the sample air and diluting it. Because the HCN meter takes 5–10 s to respond, by that time the sample air is somewhat diluted. This is one reason cup measurements are a relatively constant proportion (<1) of comparable desiccator jar measurements.

## APPENDIX 3 Description of cyanide extraction and quantification procedures.

### Cyanide extraction

The following extraction method is a modification of the protocol developed by Arias et al. (2016). Leaf material tissue was milled to a fine powder using liquid nitrogen in a 8‐cm‐diameter mortar and pestle, and the powdered material was weighed and placed in a glass scintillation vial. Total cyanogenic glycosides (CGs) were extracted by adding 0.1 M H_3_PO_4_ (4 mL) and stirring the mixture with a magnetic bar at room temperature for 1 h. The mixture was then filtered using 7‐mm‐diameter glass pipettes with cotton plugs acting as filters. To hydrolyze the cyanogenic compounds by cleaving the glycosidic bond and releasing the cyanohydrin aglycone from the glucose molecule, a 2‐mL aliquot of the filtrate was mixed with 2 mL of 5 M H_2_SO_4_ in a tightly capped scintillation vial and heated at 98°C for 1 h. The hot solution was cooled in an ice bath for 15 min, then 5 mL of cold 5 M NaOH was added to hydrolyze the cyanohydrin group and trap the liberated HCN compounds as NaCN. These alkaline solutions were left to stand on the bench at room temperature for 1 h to guarantee complete reaction of the cyanogenic compounds with the NaOH. The colorimetric method followed.

### Quantification of cyanogenic glucosides by colorimetric analysis

Quantification followed the Lambert cyanide colorimetric method (Lambert et al., 1975) and was compared with the new HCN meter quantification method presented here. Aliquots (125 μL) of the final solution were transferred to 20‐mL scintillation vials containing 0.2 M phosphate buffer (875 μL, pH 6). Then 0.4 mL of *N*‐chlorosuccinimide/succinimide (NCS) oxidizing reagent solution was added to each vial. These oxidized solutions were left to stand at room temperature for 20 min, then 1.6 mL of chromogenic pyridine/barbituric acid solution was added. Chromogenic reagents bind to the molecule of interest as a function of its concentration in solution. After 20 min, when the mixture developed a purple color, absorbance of the samples was measured using a UV‐Vis spectrophotometer (Helios β Spectronic Unicam, Thermo Fisher Scientific, Waltham, Massachusetts, USA) at 580 nm against a blank solution containing phosphate buffer and all other reagents used in the colorimetric process. NaCN concentration for each sample was calculated by comparing its absorbance with a calibration curve calculated using solutions of known NaCN concentration (*R* ^2^ = 0.99, *P* < 0.001). This curve spanned the range of NaCN concentration found in these samples. Absorbance of each sample was plotted on the *x*‐axis and known NaCN concentration was plotted on the *y*‐axis in Microsoft Excel.

HCN concentration values (μg/g dry weight) from the colorimetric method were converted to μmol/g so that units were comparable with concentration data from the meter HCN quantification method. Concentration values from the meter method are based on fresh leaf (“wet”) weight. These values were converted to dry weight using dry tissue content conversions for all *Passiflora* species sampled (C.R.M., unpublished data) to be comparable with the colorimetric data.

### Colorimetric procedure reagent preparation

#### Phosphate buffer solution preparation

Na_2_HPO_4_·2H_2_O (35.61 g) and NaH_2_PO_4_·H_2_O (27.6 g) were dissolved separately in distilled water. The volume of each stock solution was diluted to 1000 mL. These stock solutions were stored in the refrigerator at 4°C. For a 50‐mL aliquot of buffer, 38.5 mL of Na_2_HPO_4_·2H_2_O and 11.5 mL of NaH_2_PO_4_·H_2_O were mixed. The pH of the solution was adjusted to 6.0.

#### NCS preparation

Succinimide (0.25 g) was dissolved in 40 mL of distilled water. Then, 0.025 g of *N*‐chlorosuccinimide was added and mixed with a magnetic stir bar. The solution was then dissolved up to 100 mL with distilled water and stored in a paper bag in the refrigerator.

#### Pyridine/barbituric acid chromogenic reagent preparation

Two grams of barbituric acid were dissolved in approximately 10 mL of distilled water with a stir bar, and then 10 mL of pyridine was added. The solution was diluted to 50 mL with distilled water and stored in a paper bag in a refrigerator for a maximum of two weeks.

## APPENDIX 4 Procedure for calibrating the plastic cup technique.

Put out two mortar‐pestles, and weigh two plastic cups with lids on. Then roll the *Passiflora* leaves into a cylinder (we used *P. biflora*) and quickly cut a 2‐mm piece off the roll into mortar #1, then cut another piece into #2, then another into #1, alternating until the roll is finished. Crush the leaves in mortar #1 to rupture the leaf parenchyma, and place the crushed material in cup #1 and seal. Then quickly do the same with the leaves in mortar #2. Weigh the cups. Cup #1 is then analyzed following the cup protocol. Cup #2 is put in the glass container with the meter turned on; the cup lid is then opened and the glass container lid is closed before HCN gas can escape. Record data until ppm readings are asymptote. Compare the desiccator values to the plastic cup values by plotting log_10_‐transformed values into a graph and fitting a line to the data. Then calculate the fixed ratio between the methods using the slope and *y*‐intercept of the linear equation provided in Fig. 3.
