# Supplementary material for: Using a portable hydrogen cyanide gas meter to uncover a dynamic phytochemical landscape
Source: Appl Plant Sci. 2020 Apr 19;8(4):e11336. doi: 10.1002/aps3.11336 (PMC7186902; doi:10.1002/aps3.11336)
Supplement: Supplementary file 1 — APPENDIX S1. Combined mortar‐pestle (MP) device. [file APS3-8-e11336-s001.pdf]

APPENDIX S1. Combined mortar-pestle (MP) device.

Photograph of MP and component parts

The top panel shows the component parts of the MP device: borosilicate glass rod (the "pestle") (A), Teflon screw with rubber gasket (B), GL 25 internal thread (the "mortar") (C), GL 14 intake thread (D), and rubber stopper (E). The bottom panel shows the complete unit assembled to measure crushed *Passiflora* leaves. Note that this assembled unit has a manual aspirator hand pump. This hand-operated pump is interchangeable with the battery-powered sampler pump.

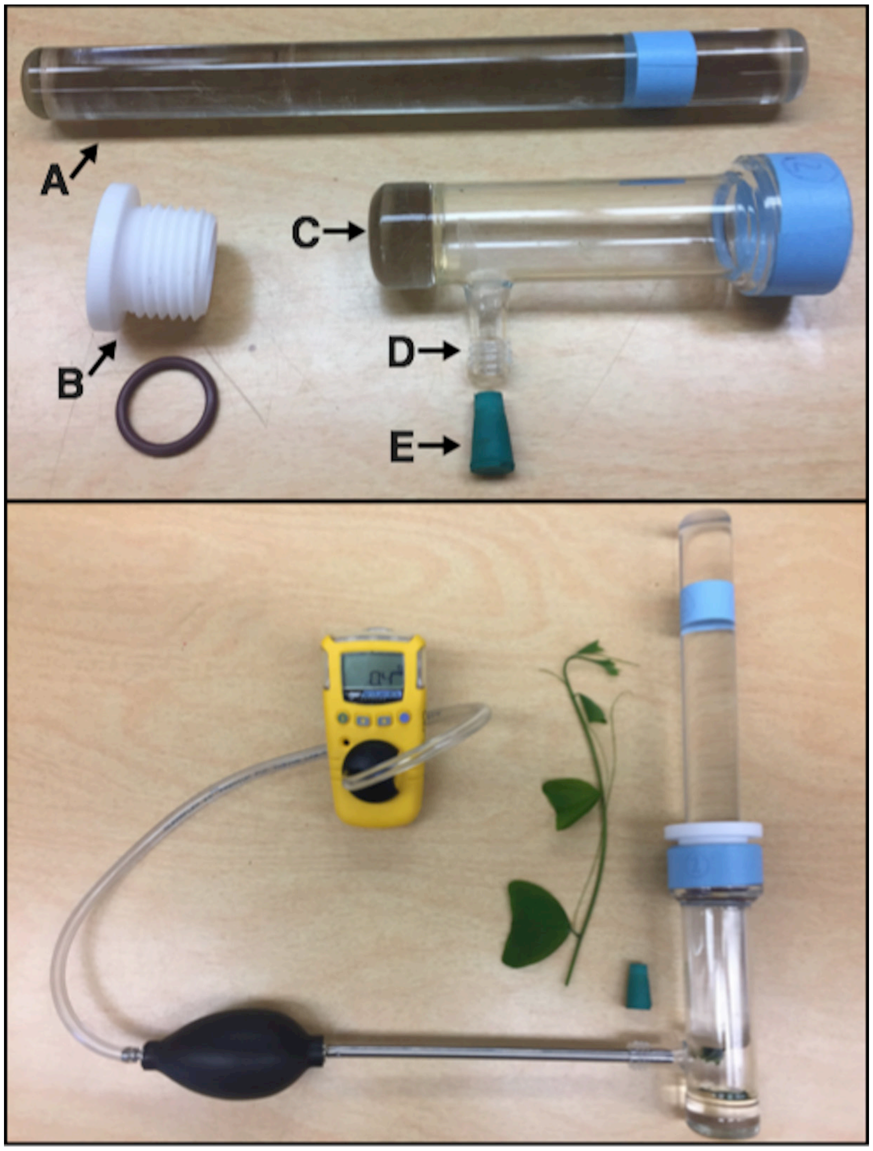

Fabrication of the MP

The apparatus consists of a 1-inch-wide borosilicate glass rod (the "pestle") that fits snugly into a GL 25 internal thread (the "mortar"). The glass rod is inserted into the center of the large-threaded Teflon screw (see B in the top panel above). The bottom of the GL thread and the glass rod were lathed so that the end of the glass rod fits snugly against the concave surface at the bottom of the chamber. A GL 14 intake thread is glass blown to the side of the GL 25; this is where a tube that is connected to the sampler pump enters the chamber and draws sample air toward the meter. A rubber stopper is placed inside the side thread prior to mastication of the plant material. Once the thread is tightened and the stopper is in place, the chamber is hermetically sealed.
