# Supplementary material for: Using a portable hydrogen cyanide gas meter to uncover a dynamic phytochemical landscape
Source: Appl Plant Sci. 2020 Apr 19;8(4):e11336. doi: 10.1002/aps3.11336 (PMC7186902; doi:10.1002/aps3.11336)

APPENDIX S2. HCN gas measurement "plastic cup" apparatus showing benchtop setup at La Selva Biological Station, Costa Rica.

Here three samples were being processed simultaneously. Milligram balance not shown. The plastic cup reaction chamber is connected with a hose to the sampler gas pump, which is connected in turn to the HCN meter by a connector that is supplied with the meter.

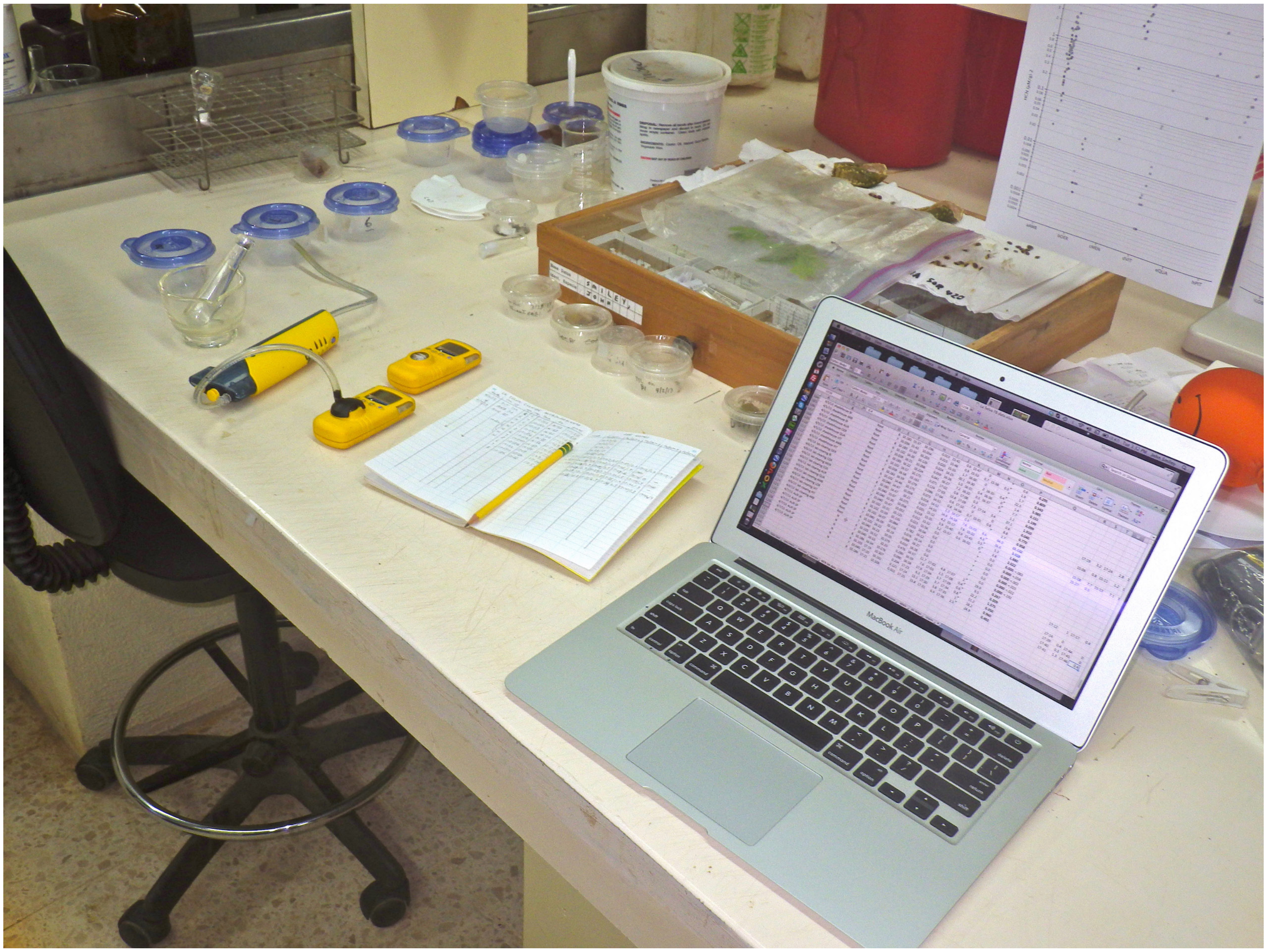

Supplement: Supplementary file 2 — APPENDIX S2. HCN gas measurement “plastic cup” apparatus showing benchtop setup at La Selva Biological Station, Costa Rica. [file APS3-8-e11336-s002.pdf]
