# Supplementary material for: Using a portable hydrogen cyanide gas meter to uncover a dynamic phytochemical landscape
Source: Appl Plant Sci. 2020 Apr 19;8(4):e11336. doi: 10.1002/aps3.11336 (PMC7186902; doi:10.1002/aps3.11336)

APPENDIX S3. Colorimetric data comparison. Data collected on the right side of the leaf midvein is regressed against data collected on the left side of the leaf. Each dot represents one individual leaf. *Passiflora* species identity is not indicated.

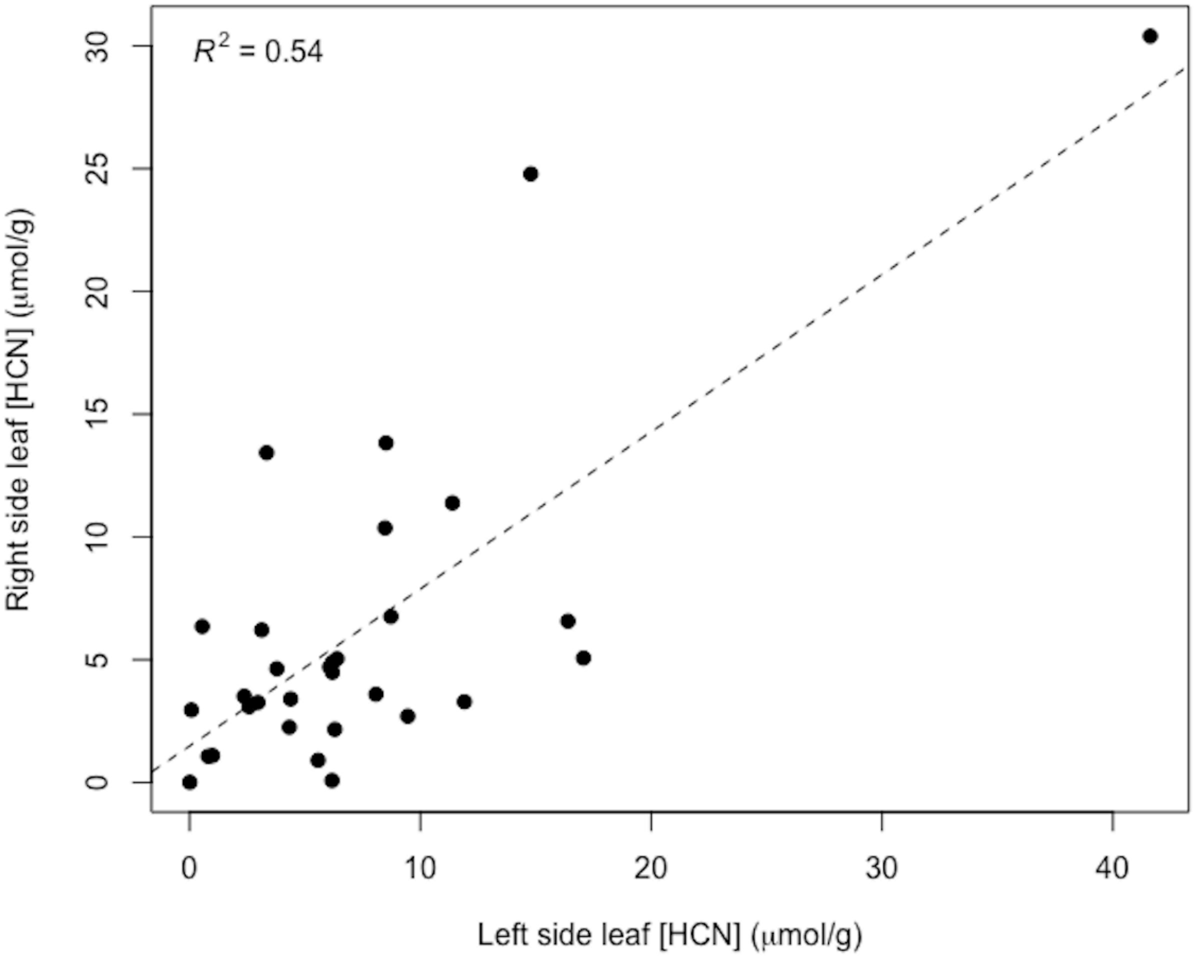

Supplement: Supplementary file 3 — APPENDIX S3. Colorimetric data comparison. [file APS3-8-e11336-s003.pdf]
