# Supplementary material for: Using a portable hydrogen cyanide gas meter to uncover a dynamic phytochemical landscape
Source: Appl Plant Sci. 2020 Apr 19;8(4):e11336. doi: 10.1002/aps3.11336 (PMC7186902; doi:10.1002/aps3.11336)
Supplement: Supplementary file 4 — APPENDIX S4. An example of several dimensions of Passiflora intraspecific HCN variation. [file APS3-8-e11336-s004.docx]

**APPENDIX S4.** The plastic cup technique can also be used to measure cyanogenesis in crushed arthropods. Insects of each species were pooled into one sample to increase the likelihood of HCN detection.

| **Species (Age/description)** | **No. of insects, pooled into one sample** | **Pooled weight (mg)** | **μmol HCN/g insect** | **Limit of detection*** |
| --- | --- | --- | --- | --- |
| *Parchicola* sp. (yellow-tibia adults) | 7 | 18 | 0 | <0.106 |
| *Monomacra violacea* (adults) | 10 | 30 | 0 | <0.064 |
| *Ptocadica bifasciata* (adults) | 5 | 37 | 0 | <0.052 |
| *Ptocadica* sp. (red adults) | 6 | 35 | 0 | <0.054 |
| *Parchicola* sp. (DF-2 adults) | 7 | 11 | 0 | <0.176 |
| *Pedilia* sp. (red adults) | 6 | 88 | 0 | <0.023 |
| *Pedilia* sp. (red larvae) | 9 | 132 | 0 | <0.015 |
| *Heliconius sara* (adults) | 2 | 68 | 0.415 |  |
| *H. sara* (larvae) | 1 | 136 | 0.892 |  |

*Calculated by entering the lowest possible reading from the detector (0.3 ppm) into the formula for calculating μmol/g. The actual value is less than the limit of detection value.
