# Supplementary material for: Using a portable hydrogen cyanide gas meter to uncover a dynamic phytochemical landscape
Source: Appl Plant Sci. 2020 Apr 19;8(4):e11336. doi: 10.1002/aps3.11336 (PMC7186902; doi:10.1002/aps3.11336)

Smiley and Morrison—Applications in Plant Sciences 2020 8(4)—Data Supplement S5
DOI

APPENDIX S5. An example of several dimensions of *Passiflora* intraspecific HCN variation.

These data showcase HCN variation within and between plants, branches, and leaves, showing HCN amounts produced by separate *Passiflora auriculata* branches (colored symbols) from widely spaced individual plants listed on the *x*-axis. The *y*-axis is plotted on a log scale.

Passiflora auriculata exhibits large variation in the amount of HCN produced, with the greatest value more than 200 times higher than the lowest value. Examining the individual plants on the *x*-axis and noting the log scale for the *y*-axis, it is evident that some plants produce fully 10 times more HCN than others (e.g., "garden" vs. "boat"). Within each plant, the greatest amount is on average about 10 times the smallest amount. Much of this variation is between branches, with some branches producing more HCN than others. "SP1c" illustrates this with the three highest readings from one branch (green box), the middle three readings from another branch (red "Y"), and the lowest three readings from a third branch (purple triangle).

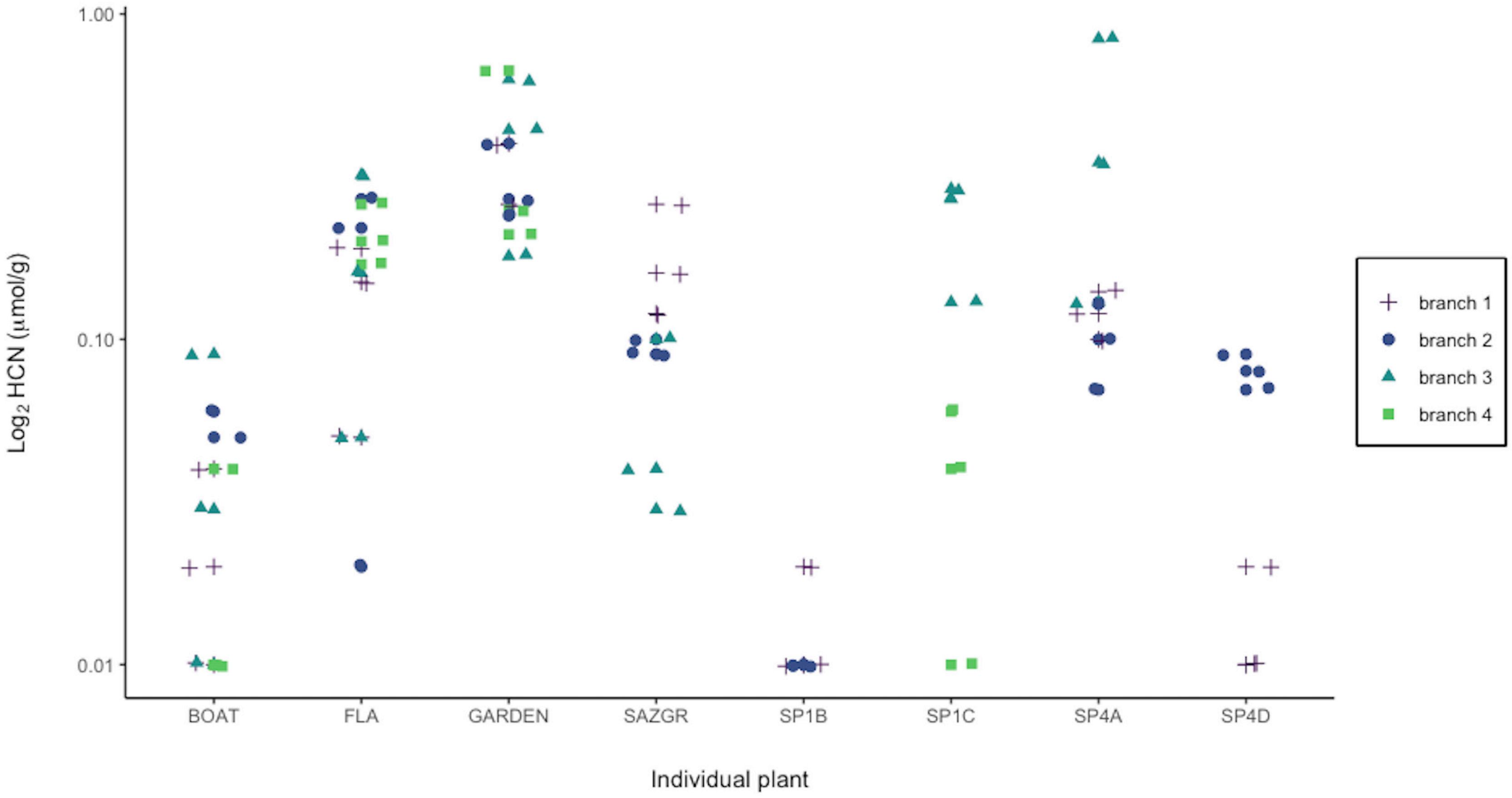

Supplement: Supplementary file 5 — APPENDIX S5. Dynamic spatial and temporal HCN variation in Passiflora biflora. [file APS3-8-e11336-s005.pdf]
