# Supplementary material for: Using a portable hydrogen cyanide gas meter to uncover a dynamic phytochemical landscape
Source: Appl Plant Sci. 2020 Apr 19;8(4):e11336. doi: 10.1002/aps3.11336 (PMC7186902; doi:10.1002/aps3.11336)

APPENDIX S6. *Heliconius doris* (Lepidoptera: Nymphalidae) feeding-induced reduction in cyanogenesis in *Passiflora ambigua*. Leaf disks were sampled from leaves with actively feeding larvae and from adjacent leaves that had not yet experienced herbivory. The HCN concentration difference between leaves with larvae and control leaves was statistically significant (t-test, df = 90, $P < 0.001$).

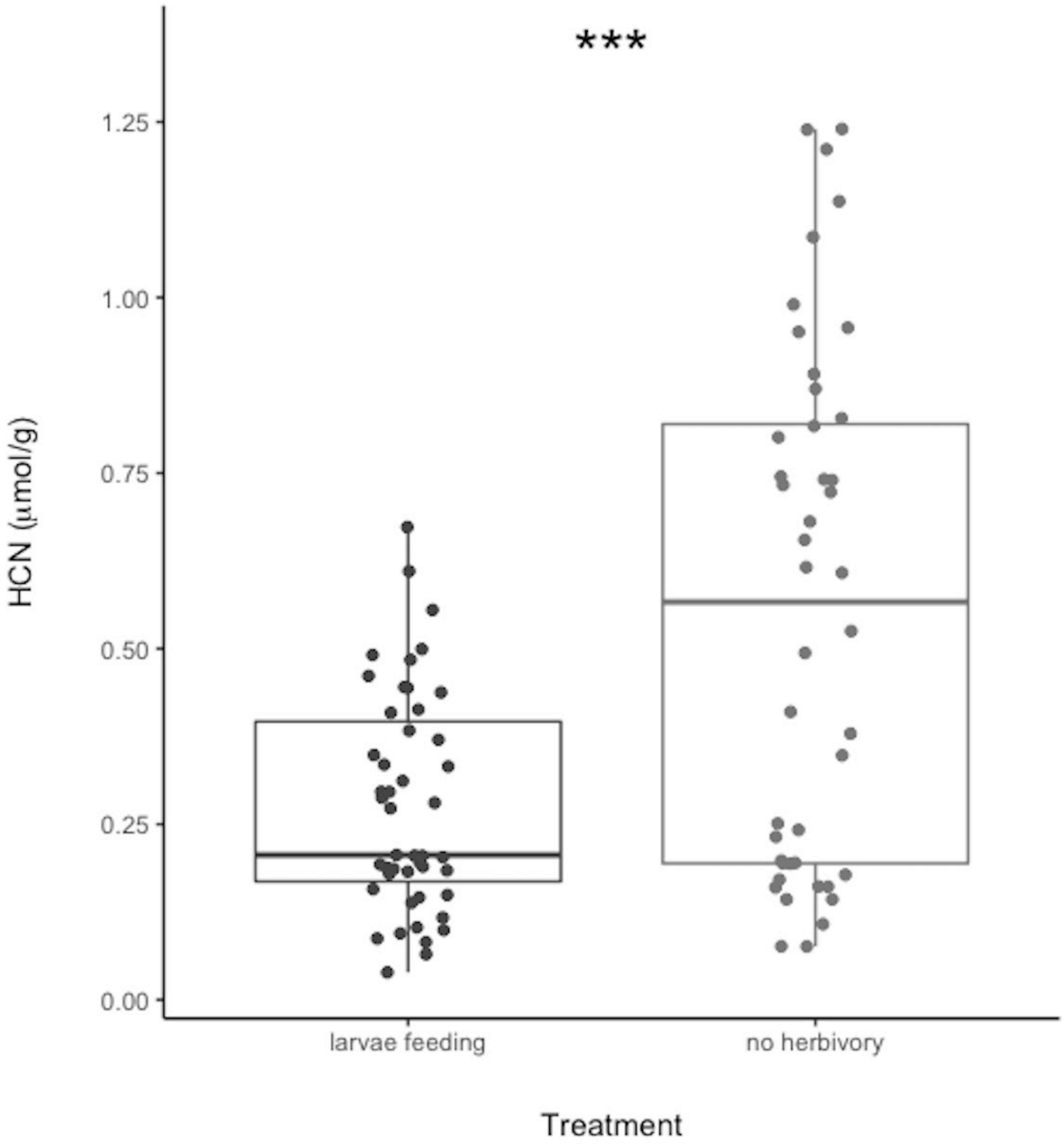

Supplement: Supplementary file 6 — APPENDIX S6. Possible within‐leaf induction of cyanogenic glycosides in Passiflora ambigua and P. auriculata. [file APS3-8-e11336-s006.pdf]
