# Supplementary material for: Using a portable hydrogen cyanide gas meter to uncover a dynamic phytochemical landscape
Source: Appl Plant Sci. 2020 Apr 19;8(4):e11336. doi: 10.1002/aps3.11336 (PMC7186902; doi:10.1002/aps3.11336)

APPENDIX S7. Dynamic spatial and temporal HCN variation in *Passiflora biflora*. (A) Distribution of *P. biflora* leaf HCN with age and sampling time. Leaves were plucked from a branch in the forest, in the order of youngest to oldest (1…20) at different times (t = 0…54 h). At time zero (t_0, red squares), there is a marked reduction in HCN in older leaves. Leaves plucked from the same branch at later times (other symbols) fall on the same steeply curving line, indicating a lack of response. Apparently, under these conditions in *P. biflora*, leaf plucking does not induce greater amounts of cyanogenic glycosides in adjacent leaves. (B) Dynamic HCN activity in *P. biflora*. This slender branch, which was found growing in the shade and was removed by cutting the stem below leaf #14, illustrated a contrasting and unexpected pattern. The branch was taken back to the lab, and three leaves were immediately analyzed (red boxes). Other leaves were then sampled at subsequent times. The result is a complex combination of partial HCN reduction on the cut branch and nearly complete elimination of HCN from the remaining branch. This suggests that HCN production for this, and perhaps other, *Passiflora* species is under rapid, active control by the plant.

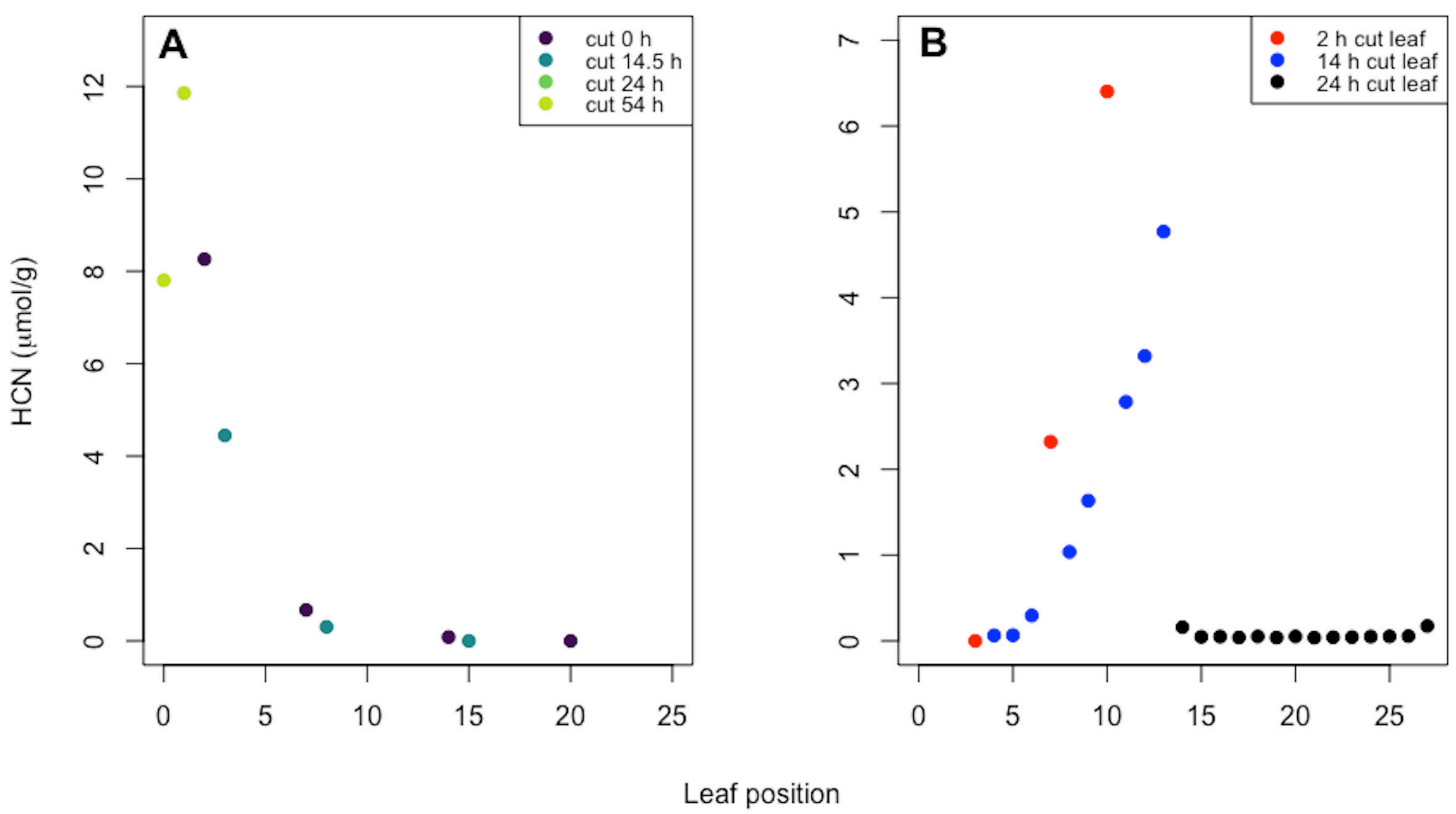

Supplement: Supplementary file 7 — APPENDIX S7. Heliconius doris (Lepidoptera: Nymphalidae) feeding‐induced reduction in cyanogenesis in Passiflora ambigua. [file APS3-8-e11336-s007.pdf]
