# Supplementary material for: Using a portable hydrogen cyanide gas meter to uncover a dynamic phytochemical landscape
Source: Appl Plant Sci. 2020 Apr 19;8(4):e11336. doi: 10.1002/aps3.11336 (PMC7186902; doi:10.1002/aps3.11336)

APPENDIX S8. Possible within-leaf induction of cyanogenic glycosides in *Passiflora ambigua* and *P. auriculata*. Odd-numbered leaves of *P. ambigua* (A) and *P. auriculata* (B) were sampled by cutting off the leaf tip at t = 0 h. At t = 24 h and at t = 48 h, these leaves were resampled and found to have increased by 50–100%. In contrast, the even-numbered leaves showed no such increase. By contrast, cutting damage to *P. auriculata* leaves did not appear to increase HCN amounts. Instead, amounts showed a tendency to decrease.

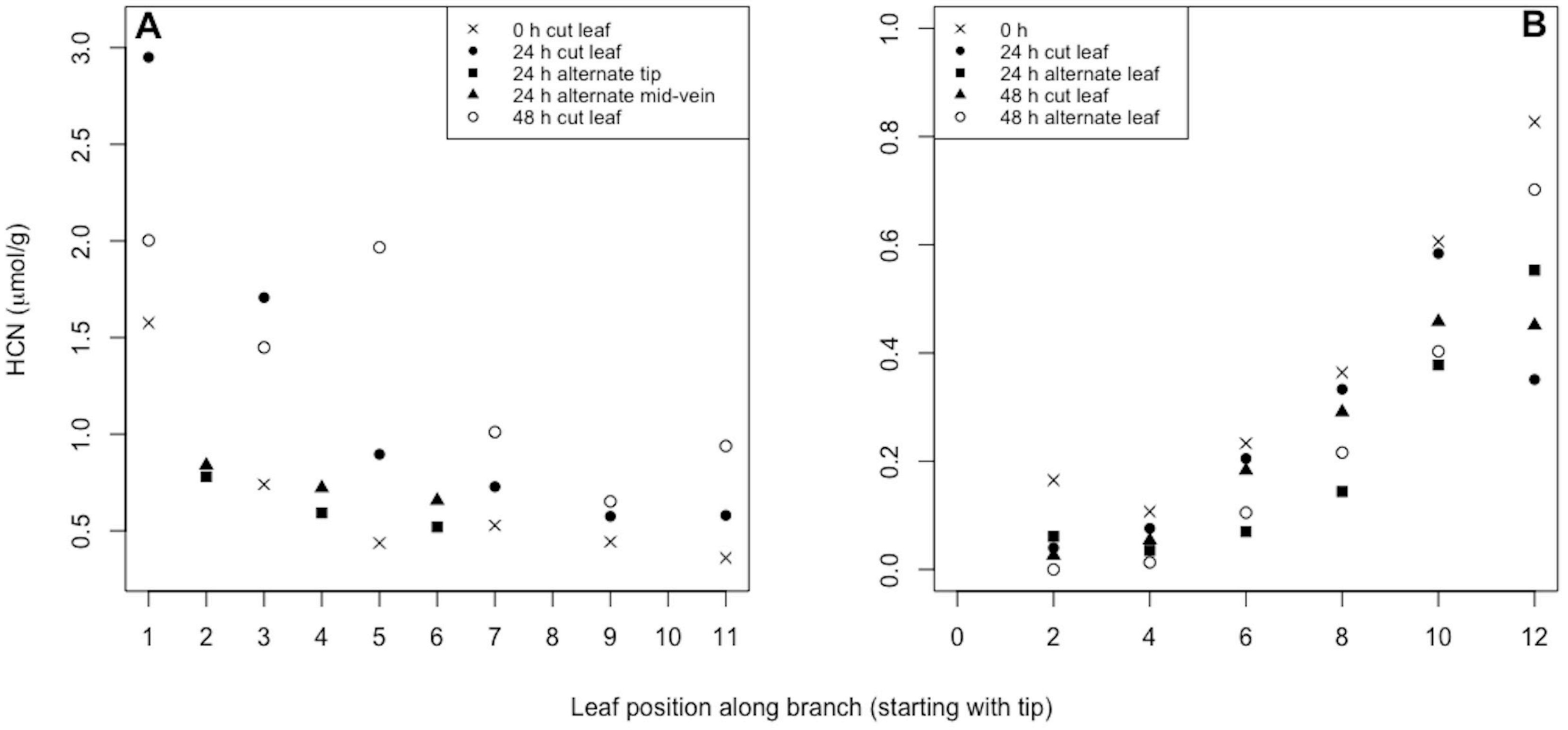

Supplement: Supplementary file 8 — APPENDIX S8. Tabulation of sample sizes from Fig. 2. [file APS3-8-e11336-s008.pdf]
