# Supplementary material for: Using a portable hydrogen cyanide gas meter to uncover a dynamic phytochemical landscape
Source: Appl Plant Sci. 2020 Apr 19;8(4):e11336. doi: 10.1002/aps3.11336 (PMC7186902; doi:10.1002/aps3.11336)
Supplement: Supplementary file 9 — APPENDIX S9. The plastic cup technique can also be used to measure cyanogenesis in crushed arthropods. Insects of each species were pooled into one sample to increase the likelihood of HCN detection. [file APS3-8-e11336-s009.docx]

APPENDIX S9. Tabulation of sample sizes from Fig. 2.

| **Species^a^** | **New leaf** | **Mature leaf** | **Old leaf** | **Stem** | **Tendril** | **Root** | **Flower** | **Fruit** |
| --- | --- | --- | --- | --- | --- | --- | --- | --- |
| *Passiflora ambigua* | 210 | 148 | 37 | 2 | 3 | 6 | — | — |
| *P. arbelaezii* | 34 | 49 | — | 2 | — | 2 | — | 5 |
| *P. auriculata* | 278 | 161 | 7 | 2 | — | 3 | — | — |
| *P. biflora* | 263 | 160 | 4 | 7 | — | 5 | 4 | 3 |
| *P. coriacea* | 43 | 31 | 8 | — | — | 1 | — | 1 |
| *P. costaricensis* | 19 | 17 | 3 | 1 | — | 2 | — | 1 |
| *P. lobata* | 5 | 3 | 1 | 3 | 1 | 2 | — | — |
| *P. menispermifolia* | 32 | 35 | 2 | 1 | — | 2 | — | 1 |
| *P. oerstedii* | 4 | 8 | 1 | 1 | — | 2 | — | 1 |
| *P. pittieri* | 57 | 43 | 10 | 4 | — | 1 | 3 | — |
| *P. quadrangularis* | 33 | 5 | — | — | — | 1 | — | — |
| *P. vitifolia* | 5 | 4 | — | 5 | 2 | 2 | — | 6 |

*Note:* — = tissue was not sampled.

^a^All *Passiflora* species at La Selva Biological Station.
